# Global emergence and γ-aminobutyric acid type A (GABA_A_) receptor activity of the new designer benzodiazepine ethylbromazolam

**DOI:** 10.1007/s00204-026-04433-9

**Published:** 2026-05-20

**Authors:** Caitlyn Norman, Dean Acreman, Meera Bissram, Blake Curtis, Sebastian Hamer, Folker Westphal, Michael Putz, Cristiana Stefan, Sarah R. Delaney, Rick Lines, Malcolm D. McLeod, Karen McDonald, Henrik Green

**Affiliations:** 1 Division of Clinical Chemistry and Pharmacology, Department of Biomedical and Clinical Science, Linköping University, Linköping, Sweden; 2 Welsh Emerging Drugs and Identification of Novel Substances Project, Public Health, Cardiff, Wales, UK; 3 Toronto’s Drug Checking Service, St. Michael’s Hospital, Unity Health Toronto, Toronto, Canada; 4 Clinical Laboratory and Diagnostic Services, Centre for Addiction and Mental Health, Toronto, Canada; 5 Research School of Chemistry, Australian National University, Canberra, ACT Australia; 6 CanTEST Health and Drug Checking Service, Canberra, ACT Australia; 7 State Bureau of Criminal Investigation Schleswig-Holstein, Forensic Science Institute, Kiel, Germany; 8 Federal Criminal Police Office, Forensic Science Institute, Wiesbaden, Germany; 9 Department of Laboratory Medicine and Pathobiology, University of Toronto, Toronto, Canada; 10 Department of Laboratory Medicine, St. Michael’s Hospital, Toronto, Canada; 11 The Australian Drug Observatory, Australian National University, Canberra, ACT Australia; 12 Department of Forensic Genetics and Forensic Toxicology, National Board of Forensic Medicine, Linköping, Sweden; 13 Science for Life Laboratory, Department of Biomedical and Clinical Science, Linköping University, Linköping, Sweden

**Keywords:** New psychoactive substances, γ-aminobutyric acid receptor type A, Designer benzodiazepine, Drug checking, Bromazolam, Ethylbromazolam

## Abstract

Designer benzodiazepines (DBZDs) are a class of new psychoactive substances (NPS) designed as legal alternatives to prescription BZDs. Bromazolam has been the most prevalent DBZD detected on the recreational market around the world; however, a new DBZD, ethylbromazolam (8-bromo-1-ethyl-6-phenyl-4 H-[1,2,4]triazolo[4,3-a][1,4]benzodiazepine; also known as bromoethylazolam) has recently emerged. In this study, the emergence of ethylbromazolam in Canada, the UK, and Australia is reported based on analysis of samples from drug checking services and in Germany based on analysis of samples seized by customs and mail services. Since November 2024, ethylbromazolam has been increasingly detected with a concurrent decrease in bromazolam detections, suggesting that its emergence is likely in response to the international control of bromazolam on 3rd December 2024. Additionally, increased detections of other DBZDs, including desalkylgidazepam (bromonordiazepam) and clobromazolam (phenazolam) have been recently observed. The in vitro α_1_β_2_γ_2_ GABA_A_ receptor activity of ethylbromazolam was determined using an automated patch clamp assay. Ethylbromazolam was found to have similar in vitro GABA_A_ receptor activity as bromazolam (EC_50_ of 10.1 nM and 15.2 nM, respectively), indicating comparable pharmacological activity and potential for harm. The market should continue to be monitored closely as it continues to evolve in response to the control of bromazolam.

**Supplementary Information:**

The online version contains supplementary material available at 10.1007/s00204-026-04433-9.

## Introduction

**Fig. 1:**
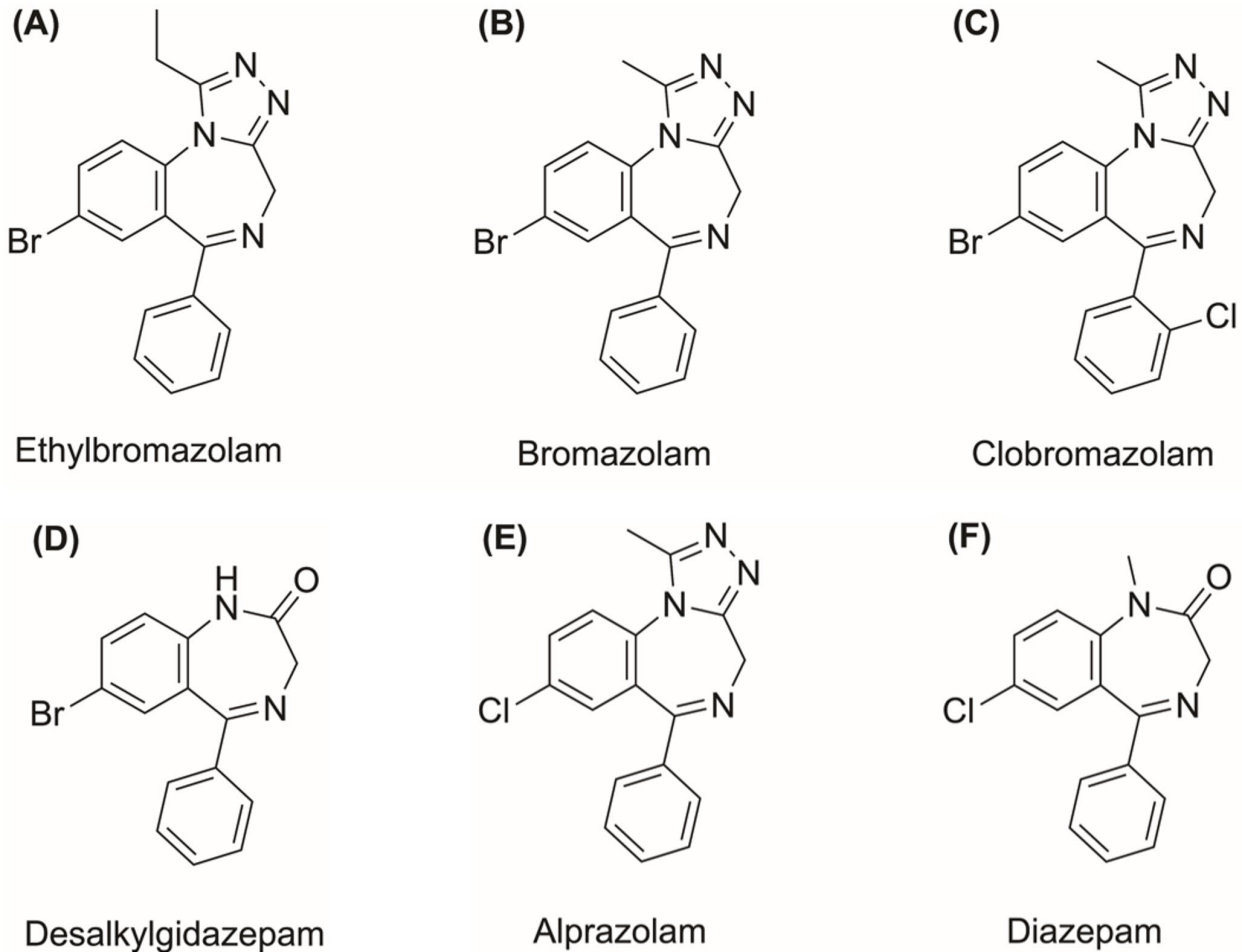
Chemical structures of the designer benzodiazepines **A** ethylbromazolam, **B** bromazolam, **C** clobromazolam, **D** desalkylgidazepam (bromonordiazepam) and other common benzodiazepines identified in this study **E** alprazolam, **F** diazepam

Benzodiazepines (BZDs) are one of the most commonly prescribed classes of drugs worldwide for conditions including anxiety, depression, insomnia, and epilepsy (McAuley et al. 2022). BZDs have been found to be prescribed in 3.2% of primary care visits and 3.8–7.4% of ambulatory care visits in the United States (Agarwal and Landon 2019; Weleff et al. 2024), but higher rates of prescription use have been reported by other countries, such as Spain (López-Pelayo et al. 2019) and Israel (Steinman et al. 2017). Long-term use of BZDs is common (Cadogan et al. 2018; Kaufmann et al. 2018; López-Pelayo et al. 2019; Murphy et al. 2016) despite prescription guidelines discouraging this due to the high risk of physical dependence and addiction (National Institute for Health and Care Excellence, 2020; The Royal Australian and New Zealand College of Psychiatrists, 2019). There are high rates of recreational use of BZDs (Schmitz 2016) with the main reasons reported as self-medication of anxiety or sleeping disorders (Wilde et al. 2021); however, they are most often used in combination with other drugs, particularly opioids and alcohol, for the reduction of unwanted effects of the other drug(s), such as withdrawal and insomnia, or enhancement of euphoric effects of the other drug(s) (Jones et al. 2012; Schmitz 2016). The use of BZDs has increasingly involved illicitly produced tablets, which are often indistinguishable from their pharmaceutical counterparts (European Monitoring Centre for Drugs and Drug Addiction (EMCDDA) 2021; McAuley et al. 2022). In the past, the majority of illicit BZD products used were diverted prescription BZDs like alprazolam (also known by the brand name Xanax; Fig. 1D) and diazepam (also known by the brand name Valium; Fig. 1E), but since 2007, the prevalence of designer BZDs (DBZDs) has been increasing (European Monitoring Centre for Drugs and Drug Addiction (EMCDDA) 2024).

DBZDs are a class of new psychoactive substances (NPS) that are designed as legal alternatives to prescription BZDs. In this paper, the term DBZDs also refers to all designer BZD-type drugs or analogs, such as the thienodiazepines (e.g., etizolam). The effects of DBZDs have been reported to be similar to prescription BZDs and include hypnosis, sedation, amnesia, anxiolysis, euphoria, loss of control, and severe withdrawal (Edinoff et al. 2022). Similar to prescription BZDs, most DBZDs are positive allosteric modulators (PAMs) of the γ-aminobutyric acid type A (GABA_A_) receptor (Batlle et al. 2018), a ligand-gated chloride channel that is the principal inhibitory receptor in the brain (Ghit et al. 2021). A recent in vitro study found the potency and efficacy of DBZDs vary greatly where the most active DBZDs were up to 60-fold more potent than diazepam. The activity of DBZDs was also found to be mostly mediated by the α^+^/g2^−^ interface, which is known as the BZD interface, on the GABA_A_ receptor; however, some DBZDs may have some activity mediated through other interfaces (Norman et al. 2026).

DBZDs are typically close analogs of prescription BZDs, where the addition or removal of methyl groups, halogens (fluorine, chlorine, or bromine), or nitro groups are the most common substitutions. Many DBZDs on the recreational market have been created from research studies or patents documenting their synthesis, such as those published by Hoffman-La Roche in the 1960s and 1970s (Field et al. 1971). Similar to other NPS, the DBZDs available on the recreational market are continuously evolving, often in response to national and international legislation. For example, etizolam was the most prevalent DBZD identified on the recreational market until its international control in November 2020 and the ban of its exportation in India in March 2021. This led to its disappearance from the market and the emergence of bromazolam, the brominated analog of alprazolam (see Fig. 1B) (Bierly et al. 2024; Dunlop et al. 2025; Ellefsen et al. 2025; Marland et al. 2024; Rodda 2024). Bromazolam has remained the most prevalent DBZD detected on the recreational market around the world; however, in March 2024, it was added to Schedule IV of the United Nation’s 1971 Convention on Psychotropic Substances, with the restriction coming into effect on 3rd December 2024 (International Narcotics Control Board, 2024). This international control of bromazolam has seemingly led to the emergence of a new DBZD, ethylbromazolam (8-bromo-1-ethyl-6-phenyl-4 H-[1,2,4]triazolo[4,3-a][1,4]benzodiazepine; also known as bromoethylazolam), a close analog of bromazolam with an ethyl group substituting the triazole ring (see Fig. 1A).

In this study, the emergence of the new DBZD ethylbromazolam is reported in four countries: Canada, the United Kingdom (UK), Australia, and Germany. In addition, the pharmacological activity of ethylbromazolam was determined using a state-of-the-art in vitro α_1_β_2_γ_2_ GABA_A_ receptor assay.

## Materials and methods

### Drug checking analysis (Toronto’s drug checking service, Canada)

Toronto’s Drug Checking Service (Toronto’s Drug Checking Service 2026) is a public health and safety program that offers timely and comprehensive unregulated drug market monitoring in Toronto, Canada. At participating community agencies, samples of drugs and used drug equipment are anonymously donated to the program by people who use drugs (i.e., service users). Each day collected samples are transported to a participating laboratory at the Centre for Addiction and Mental Health (CAMH) or St. Michael’s Hospital (SMH) where they are analyzed using gold standard mass spectrometry technologies. Analysis results are reported back to the service user along with tailored strategies to reduce harm and referrals to drug-related, health, and social services. In addition, analysis data from all samples is combined, translated, and publicly shared by the program to inform prevention, harm reduction, treatment and recovery, and community safety efforts.

Drug substances were submitted to both laboratories in pre-weighed, labelled collection vials. At CAMH, vials were re-weighed to determine sample masses and LC-MS grade methanol (Honeywell, NJ, USA) was added to achieve a solution concentration of 10 mg/mL. Samples were then crushed, vortex mixed, sonicated, and centrifuged. Following a 500-fold dilution with methanol, sample extracts were further diluted 20-fold with 15 MΩ•cm deionized water and spiked with an isotopically labelled internal standard mixture (Cayman Chemical Company, MI, USA). Analysis was conducted using a Thermo Fisher Q-Exactive high-resolution Orbitrap mass spectrometer coupled with a Vanquish UHPLC system (Thermo Fisher, Bremen, Germany).

At SMH, vials were re-weighed to determine sample masses and LC-MS grade methanol (Caledon Chemical Company, ON, Canada) was added to create solutions with a concentration of 25 mg/mL for expected BZDs or 10 mg/mL for other substances. Samples were then crushed, vortex mixed, and transferred to injection vials for analysis via electron impact GC-MS (Agilent Technologies, USA). Full details of both analytical methods used are provided in Sect. S1 of the Supplementary Information.

### Drug checking analysis (WEDINOS, UK)

The Welsh Emerging Drugs and Identification of Novel Substances Project (WEDINOS) is a drug testing service in the United Kingdom operated by Public Health Wales. The prevalence data come from 4009 publicly submitted samples that were received between 01 December 2024 and 30 September 2025. These publicly submitted sample results are available on the WEDINOS website (Welsh Emerging Drugs and Identification of Novel Substances, 2025).

Samples received were unpackaged in a fume hood, assigned a unique laboratory reference number, and weight recorded on arrival. Around 0.5 cm^3^ of the representative sample was extracted in 1 mL of LC-MS grade methanol (Rathburns, Walkerburn, Scotland). The sample was vortex mixed and diluted 1000-fold with LC-MS grade water (Rathburns) before analysis. Analysis was performed on a Waters Acquity I class UHPLC with a Waters Xevo G2-XS QTOF (Waters, Elstree, UK). Full details of the analytical method used are provided in Sect. S1 of the Supplementary Information.

### Drug checking analysis (CanTEST, Australia)

CanTEST Health and Drug Checking Service is a service operated by Directions Health Service with the assistance of the Canberra Alliance for Harm Minimisation and Advocacy and Pill Testing Australia. CanTEST is located at a single site in central Canberra, Australia that is open for 6 h per week to members of the public in possession of drugs intended for personal use. The CanTEST testing month runs from the 21st to 20th of the next month. On-site CanTEST BZD testing commenced in mid-January 2025 with the prevalence data coming from 1087 publicly submitted samples received between 21st January and mid-August 2025.

Ethylbromazolam samples were analyzed by Fourier transform infrared spectroscopy (FTIR) using using a Bruker Alpha II FTIR (Billerica, MA, USA), BZD immunoassay test strips (BTNX Rapid Response, Ontario, Canada), UPLC with a photodiode array (PDA) detector using an Acquity UPLC H-Class PLUS fitted with an Acquity UPLC-PDA detector (Waters, Milford, MA, USA), and/or UPLC-MS/MS using a Dionex UltiMate 3000 UPLC coupled to a Q Exactive Plus hybrid Quadrupole-Orbitrap MS (Thermo Fisher Scientific). The FTIR and UPLC-PDA analyses were performed as previously described in Algar et al. (Algar et al. 2024) The first ethylbromazolam detection was also analyzed with one-dimensional ^1^H nuclear magnetic resonance (NMR) spectroscopy using a Bruker Avance III HD 800 spectrometer (800.13 MHz ^1^H) under similar conditions described in Curtis et al. (Curtis et al. 2025) Full details of the analytical method used are provided in Sect. S1 of the Supplementary Information.

### Seized sample analysis (Germany)

Two shipments found to contain ethylbromazolam tablets were seized by German customs and German mail service (Deutsche Post) at the international mail center “Niederaula” in the federal state of Hesse. The shipments came via air mail from Wolverhampton, UK and were addressed to a private person in Aachen which is located in the federal state of North Rhine-Westphalia. The tablets were initially analyzed by German customs using GC with electron ionization (EI)-MS and then sent to the EU-funded structural elucidation project NETZWERK ADEBAR (NETZWERK ADEBAR 2026; Pulver et al. 2022) to complete the characterization via GC with solid state infrared spectroscopy (sIR), LC with electrospray ionization (ESI)-MS, and NMR. The method details and different spectra for ethylbromazolam can be found in Sect. S1 of the Supplementary Information.

### In vitro α_1_β_2_γ_2_ GABA_A_ receptor activity (Sweden)

The herein used protocol for GABA_A_ receptor activity profiling of BZDs using the QPatch II (Sophion Bioscience A/S, Ballerup, Denmark), an automated patch-clamp system, has been described previously (Norman et al. 2026). A Chinese hamster ovary (CHO-K1) α_1_β_2_γ_2_ GABA_A_ cell line that stably expresses the human GABA_A_ receptor (B’SYS GmbH, Witterswil, Switzerland; RRID: CVCL_C0XQ) was used. The cells were maintained in a humidified atmosphere at 37˚C and 5% CO_2_ in Ham’s F-12 nutrient mix (Thermo Fisher Scientific, Waltham, MA, USA) supplemented with 10% heat-inactivated FBS, 250 µg/mL hygromycin B, 5 µg/mL puromycin (Sigma Aldrich, Stockholm, Sweden), 1% penicillin and streptomycin (Thermo Fisher Scientific), and 100 µg/mL zeocin (InvivoGen, San Diego, CA, USA). Cells were incubated at 30˚C overnight prior to the measurement to improve expression of the receptor and increase the electrical current. To perform the assays, cells were detached using Detachin™ cell detachment solution (5 min, 37˚C; AMSBIO, Abingdon, UK), resuspended in ex-cell animal-component free (ACF) CHO medium (Merck, Darmstadt, Germany) supplemented with 25 mM HEPES, 0.04 mg/mL soybean trypsin inhibitor (Sigma Aldrich), and 100 U/mL penicillin/streptomycin, then counted. Cells were added to a lidless microtube at the QPatch II so the QPlate cell density would be about 10.5 × 10^6^ per well.

The Sophion intracellular (IC) solution 0.0.0 stock solution was prepared in ultra-high purity water with 5.374 mM calcium chloride (CaCl_2_), 1.75 mM magnesium chloride (MgCl_2_), 10 mM HEPES, 10 mM egtazic acid (EGTA), and 120 mM potassium chloride (KCl) and was filtered. On the day of the experiments, the IC solution was prepared from the stock by adding adenosine 5’-triphosphate disodium salt hydrate (Na_2_ATP; final concentration 4 mM) and then adjusting the pH to 7.20 ± 0.01 using potassium hydroxide (KOH) and the osmolarity to 310–313 mOsm with sucrose. The Sophion extracellular (EC) solution 0.0.0 stock solution was prepared in ultra-high purity water obtained using a Milli-Q water purification system with 2 mM CaCl_2_, 1 mM MgCl_2_, 10 mM HEPES, 145 mM sodium chloride (NaCl), and 4 mM KCl and was filtered. On the day of the experiments, the EC solution was prepared from the stock by adding 1 M D-(+)-glucose (final concentration 10 mM) and then adjusting the pH to 7.40 ± 0.01 using sodium hydroxide (NaOH) and the osmolarity to 305–308 mOsm with sucrose. All reagents in the EC and IC solutions were obtained from Sigma Aldrich, except sucrose (99%) was obtained from Thermo Fisher Scientific and NaCl was obtained from VWR International (Radnor, PA, USA).

The QPatch II was used to obtain data recordings of the GABA_A_ assay. QPlate 48X (Sophion Bioscience A/S), 48 well plates for multi-hole recordings with integrated flow channels for intracellular (IC) and extracellular (EC) solutions and integrated electrodes, were used. The QPlate 48X has 10 holes per measurement site, allowing for the testing of 10 cells within each well with the final recordings an average of all cells. For recordings, the QPatch first adds IC solution to the IC flow channels, followed by EC solutions to the EC flow channels. The cells, which were centrifuged and then re-suspended in EC solution, were injected and increasing suction applied to pull the cells into the holes where the cell wall was gently ruptured for whole cell recordings. Electrodes in the EC and IC flow channels recorded the electrical signal as it changed due to chloride influx from activation of GABA (Sigma Aldrich).

The assay protocols were prepared based on the details provided in “basic protocol 1” in Schupp et al. (Schupp et al. 2020). See the Supplementary Information (Sect. S1) or Norman et al. (2026) for more information. A total of 12 recordings (5 s) were performed for each cell (six test solution concentrations with three vehicle (2 µM GABA) recordings before and after the concentration series for controls). In total, 10 µL was added to the EC flow channels (5 µL applied twice, with a 3 s interval between applications). Five seconds after each application, a pipette washing step was performed. The liquid exposure duration ranged from 40 to 300 s and the total experiment duration did not exceed 4000 s.

Dilutions of diazepam (≥ 98% purity; Sigma Aldrich), bromazolam (> 98% purity; Cayman Chemical, Ann Arbor, MI, USA), and ethylbromazolam (> 98% purity; Cayman Chemical) reference standards were prepared in DMSO (Sigma Aldrich) in 96-well microtiter plates (MTP-96) with glass inserts (Sophion Bioscience A/S). The first row of the plate was a set of vehicle control (0.3% DMSO), followed by two rows each with one test compound with two sets of six increasing concentrations. The first six columns (first set of the six concentrations) were used for pre-incubation and did not contain GABA, while the last six columns (second set of the six concentrations) were used for the main application and contained 2 µM GABA. For determining the concentration-response curve, concentrations ranged between 0.96 and 3000 nM with a 1:5 dilution. To determine if the α^+^/γ_2_^–^ interfaces (BZD binding site) is the main binding site for DBZDs, the BZD antagonist, flumazenil, was used to block the BZD binding site. For these experiments, 3000 nM flumazenil (VWR International, Stockholm, Sweden) was added to both the pre-incubation and main application sets along with increasing concentrations of ethylbromazolam (0.96 and 3000 nM). Compound dilutions were individually prepared (not as a serial dilution) so that all wells contained 0.3% DMSO.

The recordings were analyzed in the Sophion Analyzer software (version 10.0.60; Sophion Bioscience A/S), followed by GraphPad Prism (Version 10.0.2). Data was normalized to an average of the first three vehicle controls from each well and then by an average of the complete set of vehicle control run on the same QPlate. The data was normalized so the baseline GABA_A_ (2 µM) activity was at an efficacy of 1. The concentration-response curves were generated and EC_50_ and E_max_ values were calculated by curve fitting via nonlinear regression (three-parameter logistic fit) with the following constraints: bottom is a constant equal to 1 and EC_50_ must be greater than 0. The compounds tested had a reduction in current at higher concentrations, leading to a bell curve; therefore, the last concentration (3 µM) was excluded from the curve fitting. Results are represented as the normalized electrical current derived from a minimum of four independent experiments (*n* ≥ 4). Brown-Forsythe and Welch ANOVA tests (α = 0.05) were performed in GraphPad Prism to compare the EC_50_ and E_max_ values between compounds.

## Results

### Drug checking and seized sample detections

#### Canada

**Fig. 2:**
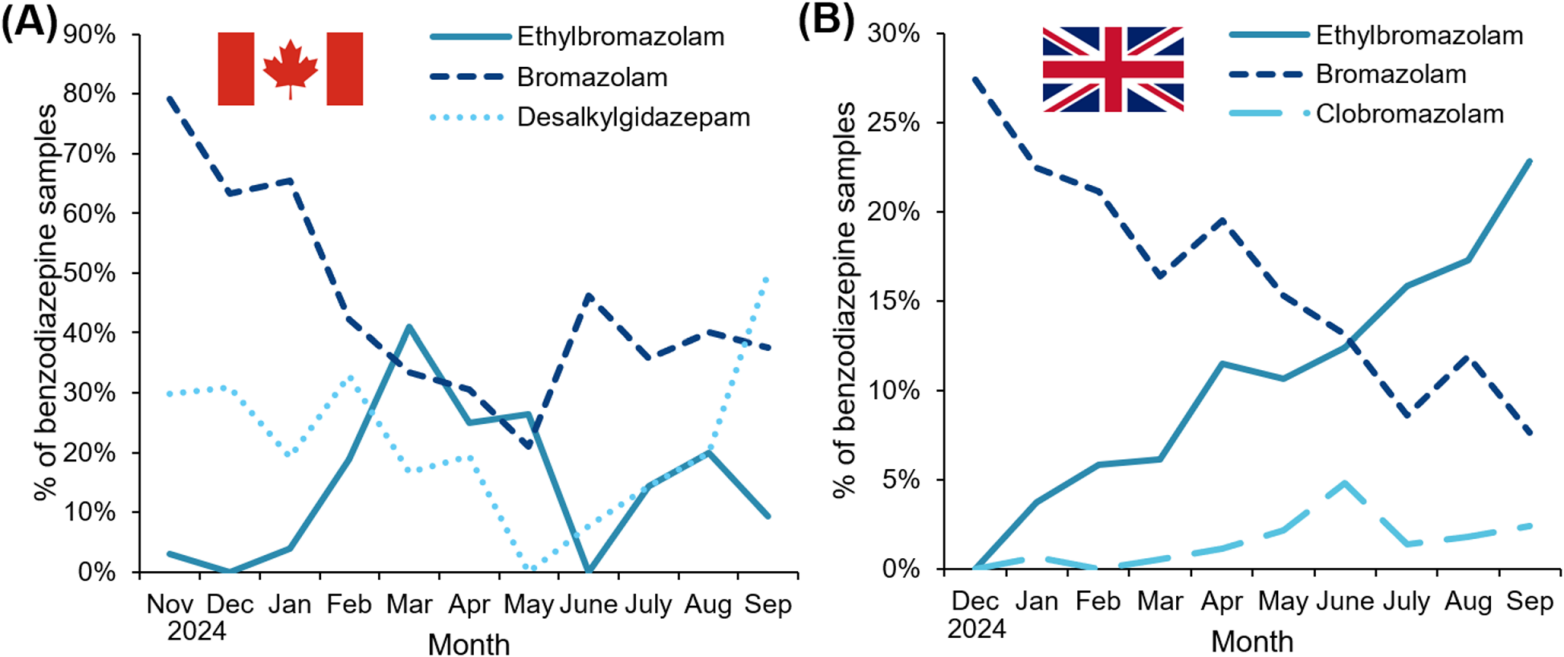
**A** Detections of ethylbromazolam, bromazolam, and desalkylgidazepam in Canada by Toronto’s Drug Checking Service between 1st November 2024 and 30th September 2025. **B** Detections of ethylbromazolam, bromazolam, and clobromazolam in the UK by WEDINOS between 1st December 2024 and 30th September 2025. Note the difference in the y-axis scale between **A** 0–90% and **B** 0–30%

Ethylbromazolam was first detected by Toronto’s Drug Checking Service in Canada on 8th November 2024. Between 1st November 2024 and 30th September 2025, there were 3148 samples examined, where 471 samples were found to contain one or more BZDs. During this time, ethylbromazolam was detected 72 times in comparison to 233 detections of bromazolam and 117 detections of desalkylgidazepam (bromonordiazepam). The percentage of BZD containing samples with detections of ethylbromazolam, bromazolam, or desalkylgidazepam is shown in Fig. 2A, demonstrating that bromazolam detections have generally been decreasing.

Ethylbromazolam was mostly found in powder samples of various colors expected by the service user to be fentanyl (*n* = 61; 84.7%), followed by alprazolam (*n* = 3; 4.2%), carfentanil (*n* = 2; 2.8%), etizolam (*n* = 1; 1.4%), xylazine (*n* = 1; 1.4%), or unknown (*n* = 4; 5.5%). This is consistent with other benzodiazepines as 82.6% of all benzodiazepine detections are in samples expected by the service user to be fentanyl.

#### United Kingdom

Ethylbromazolam was first detected in the UK by WEDINOS on 17th January 2025. Between 1st December 2024 and 30th September 2025, there were 6275 samples examined, where 2042 samples were found to contain one or more BZDs. During this time, ethylbromazolam was detected 232 times with detections increasing over time from 3.75% of BZD detections in January to 22.9% in September, as shown in Fig. 2B. In comparison, bromazolam was detected 327 times with detections decreasing over time until bromazolam was detected less than ethylbromazolam from July. Ethylbromazolam was detected alongside bromazolam in 17 samples. There was also a slight increase in clobromazolam (phenazolam) detections during this period from 1.0% to 4.8% of BZD detections. Detections of the other most commonly detected BZDs (i.e., diazepam, alprazolam, clonazepam, etizolam, lorazepam) remained relatively stable during the period.

**Fig. 3:**
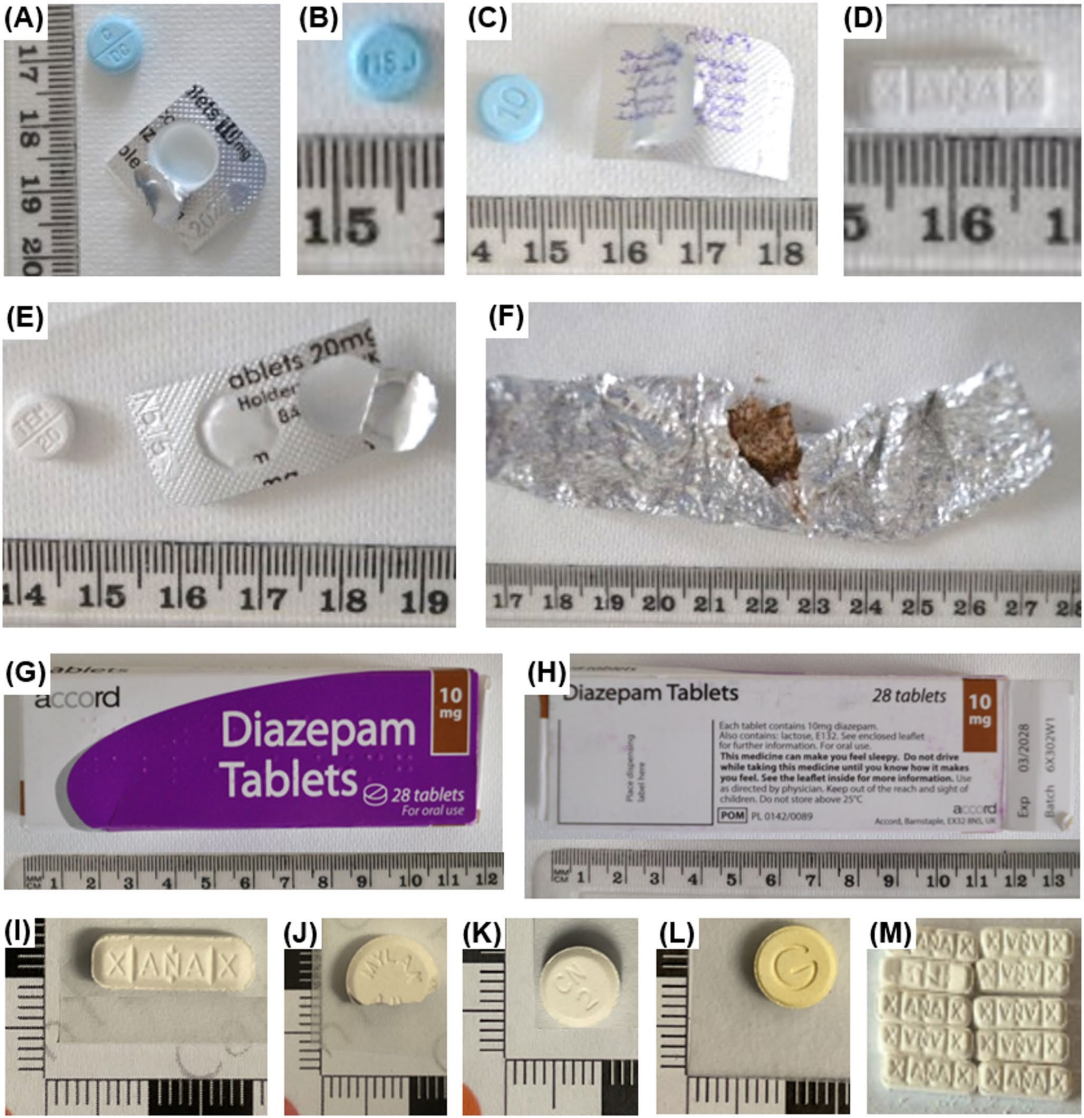
**A–H** Pictures of different samples from the UK found to contain ethylbromazolam, where **A–E** are examples of common tablet markings, **F** is a brown powder found to contain heroin and ethylbromazolam, and **G**–**H** is an example of a counterfeit diazepam prescription box where the tablets were found to contain ethylbromazolam. **I–L** Pictures of different tablet markings from samples from Australia. **M** Example of the “XANAX” tablets found to contain ethylbromazolam seized by German customs

The reported purchase intents for the samples found to contain ethylbromazolam were diazepam (67.7%; *n* = 157), alprazolam (22.4%; *n* = 52), clonazepam (2.2%; *n* = 5), unspecified BZD (2.2%; *n* = 5), heroin (2.2%; *n* = 5), zolpidem (1.3%; *n* = 3), etizolam (0.4%; *n* = 1), or temazepam (0.4%; *n* = 1) and unreported in three cases. Of the samples where heroin was the intended purchase, all but one of the samples contained opioids: one with heroin, one with protodesnitazene, one with heroin and cocaine, and one with heroin and metonitazene. A picture of the sample found to contain ethylbromazolam and heroin is shown in Fig. 3F. There was also one sample found to contain 3,4-methylenedioxymethamphetamine (MDMA).

Ethylbromazolam was mostly detected in tablets (96.6%; *n* = 224), but also as a powder (*n* = 7) and capsule (*n* = 1). Tablets were mostly blue (57.8%; *n* = 134) or white (34.5%; *n* = 80), but some were white-blue (*n* = 3), red (*n* = 3), or colorless (*n* = 2). There were three common markings for the blue tablets: C/DC (*n* = 39), MSJ (*n* = 38), and 10 (*n* = 15). Examples of these tablet markings can be found in Fig. 3A,  3B, and 3C, respectively. The white tablets were most often either round with no markings (*n* = 34) or “Xanax” bar-type tablets (*n* = 9), an example of which can be found in Fig. 3D. Two of the red tablets were also “Xanax” bar-type tablets. There was also a white tablet with the marking “TEM/20” that was sold in a blister pack as temazepam (see Fig. 3E) but was found to contain ethylbromazolam. Many tablets were found in blister packs (see Fig. 3A, 3C, and 3E for examples) and some were in counterfeit prescription boxes, an example of which can be found in Figs. 3G-H. The powders were either white (*n* = 2), white-brown (*n* = 2), or brown (*n* = 3) and the one capsule was blue.

Of the ethylbromazolam samples, 124 were submitted from England, 85 from Wales, 19 from Scotland, 3 from Northern Ireland, and 1 from an unknown region. This demonstrates the spread of ethylbromazolam through all regions of the UK. The majority of ethylbromazolam samples were submitted by males (69.8%; *n* = 162) between the ages of 16 and 78 years (mean: 39.8; median: 40). This is similar to the demographics of those submitting samples with BZDs during the same period, where 71.2% were male between the ages of 14 and 78 years (mean: 38.2; median: 37).

#### Australia

Ethylbromazolam was first detected in Australia by CanTEST on 11th April 2025. It was detected an additional seven times through 14th August 2025 with two detections in May, one in June, and four in August. During the same time period, there were 61 detections of BZDs, of which 19 were bromazolam. Ethylbromazolam was detected alone on three occasions, once with desalkylgidazepam (bromonordiazepam), once with alprazolam, once with bromazolam and desalkylgidazepam, once with bromazolam and MDMA, and once with the synthetic cannabinoid AB-MDMSBA and synthetic cathinone *N*-cyclohexylpentylone.

All detections were of tablets, which were either white (*n* = 6) or yellow (*n* = 2). Three of the samples were “Xanax” bar-type tablets, but other tablet markings included “MYLAN”, “CN 2”, and “DM 5” on one side with “G” on the other side, which can be seen in Fig. 3I-L. The reported purchase intents for these samples found to contain ethylbromazolam were alprazolam (50%; *n* = 4), diazepam (12.5%; *n* = 1), clonazepam (12.5%; *n* = 1), an unspecific BZD (12.5%; *n* = 1), or not specified (12.5%; *n* = 1).

Ethylbromazolam was not identified by FTIR, likely due to the interference of excipients and cutting agents in the samples. Six of the eight samples were tested using an immunoassay test strip, where three were negative. The three samples that were positive contained other BZDs, so it is possible the test strip was detecting the presence of the other BZDs rather than ethylbromazolam. However, an ethylbromazolam reference standard has not yet been tested with the test strips to determine its reactivity and limit of detection, so caution should be taken in interpreting these test strip results.

#### Germany

Ethylbromazolam was detected in tablets seized from two shipments on 10th March 2025 in the scope of customs investigations in the international mail centre “Niederaula”, about 130 km from Frankfurt. The first shipment consisted of 273 tablets plus some fragments of tablets that were declared as “supplements”. An associated bill stated a content of “250 tablets Contrave (naltrexone/bupropion)” with a value of 30 British pounds. The second shipment contained 1092 tablets with identical analytical data as the first shipment. Both shipments originated from Wolverhampton, UK and only contained the tablets. All tablets contained ethylbromazolam mixed with caffeine (see analytical data in the Supplementary Information) and were white rectangular tablets bearing the “XANAX” marking as shown in Fig. 3M.

### In vitro **α**_**1**_**β**_**2**_**γ**_**2**_**GABA**_**A**_**receptor activity**

**Fig. 4:**
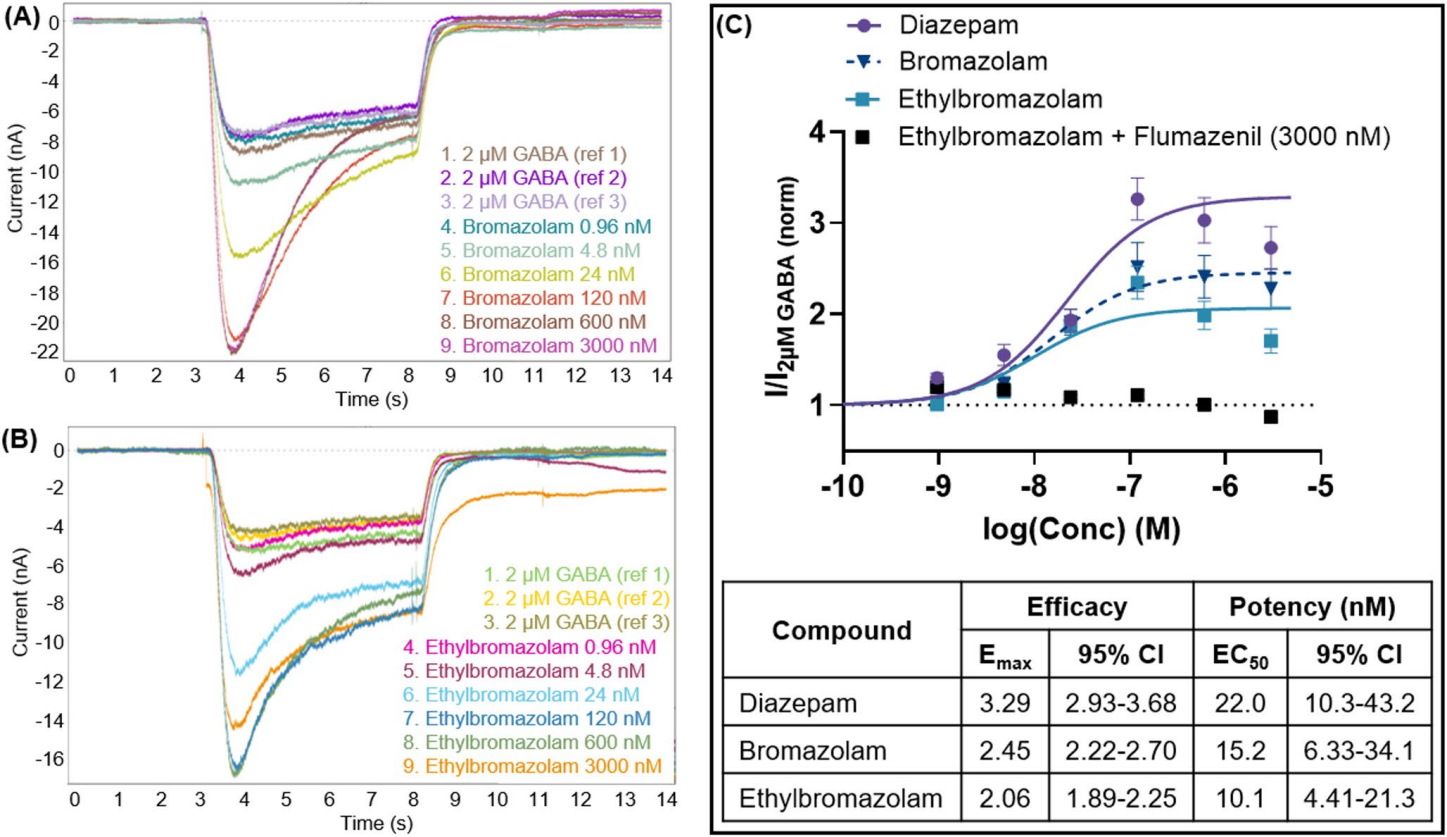
The electrical current from one cell generated in this study for **A** bromazolam and **B** ethylbromazolam. **C** The concentration-response curves and calculated efficacy (E_max_) and potency (EC_50_) values for diazepam, bromazolam, and ethylbromazolam from a minimum of four independent experiments (*n* ≥ 4). Ethylbromazolam in the presence of the BZD antagonist flumazenil (3000 nM) is also included. The data was normalized so the baseline GABA (2 µM) activity was at an efficacy of 1, which is shown as a dotted line. Error bars indicate standard error to the mean (SEM)

The electrical current from one cell generated from the in vitro α_1_β_2_γ_2_ GABA_A_ receptor assay for ethylbromazolam and bromazolam are shown in Fig. 4A and B, respectively. This demonstrates the clear increase in chloride flux with increasing concentrations of the DBZDs. The resulting concentration-response curves and calculated efficacy (E_max_) and potency (EC_50_) values are provided in Fig. 4C. Ethylbromazolam had an efficacy of 2.06 and a potency of 10.1 nM, which were not significantly different from those of bromazolam. In addition, the activity of ethylbromazolam was found to be completely antagonized by the BZD specific antagonist flumazenil, indicating its activity is largely mediated through the BZD α^+^/γ_2_^–^ interface.

## Discussion

Since November 2024, ethylbromazolam has been increasingly detected internationally, including in Canada, the UK, Australia, and Germany as reported in this study. Its detection has also been reported in New Zealand (High Alert, 2025) and the United States (NPS Discovery, 2025). The rapid detection of ethylbromazolam followed by notification to the public and people who use drugs of its emergence (Cantestcbr, 2025; Drugchecking, 2025) demonstrates the value of drug checking services, like Toronto’s Drug Checking Service, WEDINOS, and CanTEST, for the near-real time tracking of the recreational drug market.

The increase in ethylbromazolam and concurrent decrease in bromazolam over time, as depicted in Fig. 2A-B for Canada and the UK, suggests that its emergence is likely in response to the international control of bromazolam, which came into effect on 3rd December 2024 (International Narcotics Control Board, 2024). While ethylbromazolam is now the most prevalent DBZD detected by WEDINOS in the UK, it is not yet clear if it will become the most prevalent DBZD globally, as there have also been increased detections of other DBZDs, including desalkylgidazepam and clobromazolam, and detections of other new DBZDs, including deschlorodemethyldiazepam, ethylflualprazolam, and desmethylflutiazepam (Toronto’s Drug Checking Service, 2026). Therefore, the market should continue to be monitored closely as it continues to evolve in response to the control of bromazolam. In addition, for harm reduction purposes, people who use drugs should be informed of the current fluctuations in the DBZD market and that new BZDs may be present in their tablets despite similar markings.

Ethylbromazolam was typically detected in tablets with similar markings as other prescription and/or illicitly produced benzodiazepines from the region in the UK, Australia, and Germany. For example, many ethylbromazolam tablets in the UK had the marking “MSJ”, which is a mark typically associated with diazepam tablets marketed by MSJ Industries, a Sri Lankan generic drug manufacturer (Public Health Nigeria, 2020; Substance Misuse Resources 2026) and was also found to be a common marking for bromazolam tablets seized from the Scottish prisons (Marland et al. 2024). The markings found on the tablets in Australia (Fig. 3I-L) emulate those used by the pharmaceutical company Viatris, formerly known as Mylan Pharmaceuticals Inc, where the “MYLAN” marking emulates their alprazolam tablets (Drugs.com, 2025), the “CN 2” marking emulates their clonazepam 2 mg tablets marketed under the brand name Paxam™ (Healthdirect Australia, n.d.; Viatris, 2025), and tablets with “DM 5” on one side and “G” on the other emulate their diazepam 5 mg tablets marketed under the brand name Antenex™ (NPS MedicineWise, 2024). This use of similar tablet markings and in some cases detailed counterfeit packaging (Figs. 3G-H) indicates increased risk to people who use drugs as they are often unaware of the actual DBZD they are taking. This can be seen clearly from the reported purchase intents of those submitting samples to the drug checking services in Canada, the UK, and Australia, where a different BZD or other drug (e.g., fentanyl, zolpidem, heroin) was expected in all cases where the purchase intent was specified. This means people who use recreational drugs are typically unaware of the identity and pharmacological activity of the drugs they are taking or proper dosing.

In this study, ethylbromazolam was found to have similar in vitro α_1_β_2_γ_2_ GABA_A_ receptor activity as bromazolam. Although there is no other data available on the pharmacology of ethylbromazolam or effects, reports posted on Reddit from people who use drugs have indicated that ethylbromazolam has similar potency and dosing to bromazolam (screenshots available in Sect. S7 of the Supplementary Information) (*Do We Have Any Type of Comparison on Dosages of Ethylbromazolam Yet?*, 2025; *Ethylbromazolam / Bromoethylazolam Is by Far the #1 RC Right Now*, 2025; *Ethylbromazolam Experience*, 2025), which is consistent with the in vitro findings of this study. Though complicated by a range of factors, including bioavailability and metabolism, this indicates that ethylbromazolam is likely to produce similar effects as bromazolam and other DZBDs and has the same potential for adverse effects, including reduced consciousness, airway compromise, agitation, seizures, and myocardial injury (Bierly et al. 2024; Dunlop et al. 2025; Ehlers et al. 2024), and contributing to death (Ellefsen et al. 2025; Hikin et al. 2024; Mérette et al. 2023; Rodda 2024).

## Conclusion

Ethylbromazolam is a new DBZD that has emerged in response to the international control of its close analog bromazolam. Its detection by drug checking services in Canada, the United Kingdom, and Australia and seized samples in Germany demonstrates its international proliferation on the recreational drug market. The in vitro α_1_β_2_γ_2_ GABA_A_ receptor activity of ethylbromazolam was found to be similar to bromazolam, so it is likely to produce similar effects and have a similar harm potential.

It is recommended that clinical and forensic toxicologists, as well as chemists and drug checking services, add ethylbromazolam to their targeted and semi-targeted analytical methods. As the DBZD market is continuing to evolve, they should remain vigilant to the emergence of other new DBZDs or the increased prevalence of known DBZDs like desalkylgidazepam and clobromazolam. Public health and harm reduction services should also inform people who use drugs of the proliferation of ethylbromazolam and that the DBZD market is currently in flux so they should use appropriate caution with new purchases.

## Supplementary Information

Below is the link to the electronic supplementary material.

Supplementary Material 1

## Data Availability

All data supporting the findings of this study are available within the paper and its Supplementary Information.
