# Supplementary material for: Global emergence and γ-aminobutyric acid type A (GABAA) receptor activity of the new designer benzodiazepine ethylbromazolam
Source: Arch Toxicol. 2026 May 20;100(9):4065–76. doi: 10.1007/s00204-026-04433-9 (PMC13453982; doi:10.1007/s00204-026-04433-9)
Supplement: Supplementary file 1 [file 204_2026_4433_MOESM1_ESM.docx]

**Global emergence and γ-aminobutyric acid type A (GABA_A_) receptor activity of the new designer benzodiazepine ethylbromazolam**

Caitlyn Norman^1,*^, Dean Acreman^2^, Meera Bissram^3,4^, Blake Curtis^5,6^, Sebastian Hamer^7^, Folker Westphal^7^, Michael Putz^8^, Cristiana Stefan^3,4,9^, Sarah R. Delaney^3,9,10^, Rick Lines^2^, Malcolm D. McLeod^5,11^, Karen McDonald^3^, Henrik Green^1,12,13^

^1^Department of Biomedical and Clinical Science, Division of Clinical Chemistry and Pharmacology, Linköping University, Linköping, Sweden

^2^Welsh Emerging Drugs and Identification of Novel Substances Project, Public Health, Wales, UK

^3^Toronto’s Drug Checking Service, St. Michael’s Hospital, Unity Health Toronto, Toronto, Canada

^4^Clinical Laboratory and Diagnostic Services, Centre for Addiction and Mental Health, Toronto, Canada

^5^Research School of Chemistry, Australian National University, Canberra, Australian Capital Territory, Australia

^6^CanTEST Health and Drug Checking Service, Canberra, Australian Capital Territory, Australia

^7^State Bureau of Criminal Investigation Schleswig-Holstein, Forensic Science Institute, Kiel, Germany

^8^Federal Criminal Police Office, Forensic Science Institute, Wiesbaden, Germany

^9^Department of Laboratory Medicine and Pathobiology, University of Toronto, Toronto, Canada

^10^Department of Laboratory Medicine, St. Michael’s Hospital, Toronto, Canada

^11^The Australian Drug Observatory, Australian National University, Canberra, Australian Capital Territory, Australia

^12^Department of Forensic Genetics and Forensic Toxicology, National Board of Forensic Medicine, Linköping, Sweden

^13^Department of Biomedical and Clinical Science, Science for Life Laboratory, Linköping University, Linköping, Sweden

*Corresponding author: [caitlyn.norman@liu.se](mailto:caitlyn.norman@liu.se)

**Supplementary Information**

**SECTION 1: Method details**

**SECTION 2: Data from Toronto’s Drug Checking Service (Canada)**

**SECTION 3: Data from WEDINOS (UK)**

**SECTION 4: Data from CanTEST (Australia)**

**SECTION 5: Analytical results of ethylbromazolam sample from ADEBAR (Germany)**

**SECTION 6:** **Statistical analysis results**

**SECTION 7: Screenshots of Reddit posts about ethylbromazolam**

**SECTION 1**

**Method Details**

1. **Drug checking analysis (Toronto Drug Checking Service, Canada)**

At the Centre for Addiction and Mental Health (CAMH), samples were analyzed using a Q-Exactive Orbitrap mass spectrometer coupled with a Vanquish UHPLC system (Thermo Fisher Scientific, Bremen, Germany), utilizing both positive and negative heated electrospray ionization. Chromatographic separation was conducted on a Kinetex® F5 analytical column (2.1 × 100 mm, 2.6 μm, Phenomenex) kept at 30 °C throughout the analysis. The mobile phase consisted of water (A) and methanol (B), each containing 10 mM ammonium formate. The gradient was set at 2% B from 0 to 2.5 minutes, increased to 98% B by 13.5 minutes, and held until 14.5 minutes at a flow rate of 0.5 mL/min. Subsequently, 98% B was maintained from 14.51 to 14.95 minutes at 1 mL/min, and finally from 14.951 to 15.3 minutes at 0.5 mL/min. An injection volume of 10 µL was employed. The source conditions were as follows: capillary temperature at 325 °C, auxiliary gas heater temperature at 438 °C, auxiliary and sheath gas flow rates at 14 and 53 arbitrary units, respectively, and a spray voltage of 4.0 kV. Full scan MS data was acquired over the m/z range 75-800 with a resolution of 70,000. Stepped normalized collision energies of 30, 70, and 100 were utilized for data-dependent acquisition at a resolution of 17,500. Data processing was performed using Thermo Fisher TraceFinder software. Compound identifications were made by comparing retention time, exact mass, and fragmentation patterns to an in-house library containing approximately 1,100 compounds.

At St. Michael’s Hospital (SMH), samples were analyzed using a 6890N GC coupled with a 5975 MSD (Agilent Technologies, USA), featuring electron impact ionization. Analytes were separated on an HP-1 column (12 m × 0.2 mm × 0.33 µm) (Agilent, ON, Canada). The initial oven temperature was set at 100 °C and maintained for 3 minutes, followed by a temperature ramp of 30 °C/min to 310 °C, where it was held for an additional 5 minutes, resulting in a total run time of 15 minutes. Sample extracts of 1 µL were injected in splitless mode, utilizing helium as the carrier gas at a flow rate of 1.1 mL/min. The inlet and transfer line temperatures were both set to 280 °C. Agilent Technologies ChemStation software facilitated spectral comparison against three libraries, containing over 20,000 entries, including the Cayman Spectral library.

2. **Drug checking analysis (WEDINOS, UK)**

Samples received were unpackaged in a fume hood, assigned a unique laboratory reference number, and weight recorded on arrival. Sampling of the material was performed in a Circulation 900 fume cabinet (Monmouth Scientific, Somerset, UK). Around 0.5 cm^3^ of the representative sample was extracted in 1 mL of LC-MS grade methanol (Rathburns, Walkerburn, Scotland). The sample was vortex mixed and diluted 1000-fold with LC-MS grade water (Rathburns) before analysis. Analysis was performed on a Waters Acquity I class UHPLC with a Waters Xevo G2-XS QTOF (Waters, Elstree, UK). The mobile phases used were (A) ammonium formate (5 mmol/L, pH 3.0) and (B) acetonitrile (0.1% formic acid). The gradient used was 87:13 A:B from 0 to 10 mins; 50:50 A:B from 10 to 10.75 mins; 5:95 A:B from 10.75 to 12.5 mins; and 87:13 A:B from 12.5 to 15 mins. The flow rate was 0.4 mL/min and 0.1 μL of the dilute extract was injected onto an Acquity UPLC HSS C18 1.8 µm 2.1 × 150 mm column at 50°C. If no compounds were detected using a 0.1 μL injection, the analysis was repeated using a 10 μL injection of the diluted extract. Eluent was ionized using positive electrospray ionization (capillary voltage 0.8 kV) and cone voltage 25 V. The experiment was run in MSe mode with scanning between m/z 50 to 1000 and a collision energy ramp of 10 to 40 eV over 15 minutes against Leucine Enkephalin MS reference compound. Data obtained was compared to compounds in the UNIFI library and identification verified using retention time, exact mass, and fragmentation data for each compound.

3. **Drug checking analysis (CanTEST, Australia)**

All solvents and formic acid used throughout sample preparation and analysis were LC-grade from Thermo Fisher Scientific (Scoresby, Australia). Deuterated solvents were supplied by Cambridge Isotope Laboratories Inc. (MA, United States).

The FTIR analysis was performed using a Bruker Alpha II FTIR (Billerica, MA, USA) equipped with a diamond attenuated total reflectance (ATR) stage and zinc selenide optics as previously described in Algar et al.^26^ One whole pill was homogenized by crushing and mixing in a mortar and pestle. Following cleaning and a background scan, approximately 1-2 mg of the sample powder was loaded on the instrument for analysis.

For analysis with the immunoassay test strips, sample homogenate was prepared in 97:3 water:methanol in a 2 mL glass sample vial to a final concentration of ~1.0 mg/mL. One test strip was applied to the solution for 15 s and then placed on a non-absorbent surface to develop. After 60 s, the results were recorded as either positive (one line in the control region), negative (two lines: one in the control region and one in the test region, where even faint lines were considered negative), or invalid (no control line present).

The UPLC-PDA analysis was conducted as previously described in Algar et al.^26^ with minor alterations. Samples were prepared by applying 500 µL of 75:25 methanol:water to ~10 mg sample (powder or pill homogenate) in an Eppendorf tube. Following vortex mixing and settling, supernatant was transferred into a separate vial, diluted two-fold in 97:3 water with 0.1% formic acid:methanol, and filtered through a 0.2 mm syringe filter. Analysis was performed on an Acquity UPLC H-Class PLUS fitted with an Acquity UPLC-PDA detector (210 to 400 nm at 20 points per second) obtained from Waters (Milford, MA, USA). The mobiles phases used were (A) water with 0.1% formic acid and (B) methanol. The gradient used was 60:40 A:B from 0.0 to 6.0 mins; 10:90 A:B from 6.0 to 6.1 mins; and 97:3 A:B from 6.1 to 8.5 mins. The flow rate was 0.5 mL/min and 1 μL of the dilute extract was injected onto an Acquity UPLC CSH C_18_ column (50 × 2.1 mm, 1.7 µm) at 40˚C. The UPLC chromatogram and PDA spectrum for an ethylbromazolam containing sample are presented in Figure S4.1.

The sample prepared for UPLC-PDA analysis was transferred to a Dionex UltiMate 3000 UPLC coupled to a Q Exactive Plus hybrid Quadrupole-Orbitrap MS (Thermo Fisher Scientific) for analysis. The mobile phases used were (A) water with 0.1% formic acid and (B) methanol with 0.1% formic acid. The gradient used was 95:5 A:B from 0.0 to 1.0 min; 80:20 A:B from 1.0 to 7.0 mins; 40:60 A:B from 7.0 to 9.0 mins; 5:95 A:B from 9.0 to 11.0 mins; and 95:5 A:B from 11.0 to 12.5 mins. The flow rate was 0.5 mL/min and 1 μL of the dilute extract was injected onto a Waters Acquity UPLC CSH C_18_ column (50 × 2.1 mm, 1.7 µm) at 40˚C. Eluent was ionized using positive electrospray ionization (ESI). The experiment was run with scanning between m/z 70 to 600 and higher-energy collisional dissociation (HCD) fragmentation of 30 eV. Data were analyzed using the XCalibur (version 4.0.27.13) and Thermo FreeStyle (version 1.7.73.12) software and compared to the HighResNPS MS spectral repository. The high resolution MS/MS spectrum for ethylbromazolam is presented in Figure S4.2.

The first ethylbromazolam detection was also analyzed with nuclear magnetic resonance (NMR) spectroscopy. The sample prepared for UPLC-PDA analysis was dried down under a flow of nitrogen. It was reconstituted in ~800 µL of deuterated chloroform (Cambridge Isotope Laboratories Inc., MA, United States). One-dimensional ^1^H NMR spectra were acquired at 298 K on a Bruker Avance III HD 800 spectrometer (800.13 MHz ^1^H) equipped with a 5 mm TCI cryoprobe under similar conditions described in Curtis et al.^27^ Analysis of the spectra was completed using MestReNOVA (version 14.2.1).

4. **Seized sample analysis (Germany)**

**Gas chromatography-electron ionization-mass spectrometry (GC-EI-MS)**

Finnigan TSQ 8000 triple stage quadrupole mass spectrometer coupled to a gas chromatograph (Trace GC Ultra, Thermo Electron, Dreieich, Germany). Sample introduction: using a CTC CombiPAL (CTC Analytics, Zwingen, Switzerland) autosampler. Software: Xcalibur 4.0. For sample preparation, tablet homogenisate was suspended in Chloroform and sonicated for 5 min, then filtered and diluted for analysis. GC parameters: injection volume: 0.5 µL, splitless; injector temperature: 280 °C; carrier gas: helium; flow rate: 1.2 mL/min. MS parameters: ionization mode: EI = 70 eV; emission current: 200 µA; ion source temperature: 175 °C; scan time: 1 s; scan range: m/z = 29 – 600. Chromatographic conditions: fused silica capillary DB-1 column (30 m x 0.25 mm, film thickness 0.25 µm); temperature program: 80 °C, held for 1 min, followed by a ramp to 280 °C at 15 °C/min, held for 21 min; GC-MS transfer line: 280 °C. The GC and EI-MS spectra of ethylbromazolam are presented in Figures S5.1 and S5.2, respectively.

**Gas chromatography solid-state infrared spectroscopy (GC-sIR)**

GC-solid phase-IR-system consisting of an Agilent GC 7890B (Waldbronn, Germany) with an Agilent G4567A probe sampler and a DiscovIR-GCTM (Spectra Analysis, Marlborough, MA, USA). Sample preparation: According to GC-EI-MS without dilution. The column eluent was cryogenically accumulated on a spirally rotating ZnSe disk cooled by liquid nitrogen. IR spectra were recorded through the IR-transparent ZnSe disk using a nitrogen-cooled MCT detector. The temperature program was chosen analogous to the temperature program of GC-EI-MS analysis. GC parameters: injection: 1 µL, splitless mode; injection port temperature: 240 °C; carrier gas: helium; flow rate: 2.5 mL/min. Infrared conditions: disc temperature: -40 °C; dewar cap temperatures: 35 °C; vacuum: 0.2 mTorr; disc speed: 3 mm/min; spiral separation: 1 mm; wavelength resolution: 4 cm-1; IR range: 650-4000 cm-1; acquisition time: 0.6 s/file; 64 scans/spectrum. Data were processed using GRAMS/AI Ver. 9.1 (Grams Spectroscopy Software Suite, Thermo Fischer Scientific, Dreieich, Germany) followed by implementation of the OMNIC Software, Ver. 7.4.127 (Thermo Electron Corporation, Dreieich, Germany). Chromatographic conditions: fused silica capillary DB-1 column (30 m x 0.25 mm, film thickness 0.25 µm); temperature program: 80 °C, held for 1 min, followed by a ramp to 280 °C at 15 °C/min, held for 21 min; GC-MS transfer line: 280 °C; oven temperature: 280 °C; restrictor temperature: 280 °C. The GC-sIR spectrum of ethylbromazolam is presented in Figures S5.3 and S5.4.

**LC-ESI-linear ion trap-MS/MS**

Thermo Accela 1250 HPLC chromatograph coupled to a Thermo Velos Pro (linear trap) spectrometer with electrospray ionization; software: XCalibur 4.0. Recording of collision spectra was accomplished via direct insertion using a syringe pump. Mobile phases: Methanol with 0.0025 % formic acid (spray stabilizer), Sample Solvent: Methanol, Concentration: 10 µg/mL (if applicable), Flow rate pump: 100 µL/min, Flow rate syringe: 10 µL/min, Mass range: Automatic dynamic range (depending on molecular weight), Isolation width: 1.2 (m/z), Collision gas: Helium, Collision energy: 20 V - 60 V. Acquisition of the collision spectra was performed without and with wideband excitation. In each case, the spectrum which shows the molecular ion with nearly 10% of the base peak intensity was chosen. The LC-ESI-MS spectra of ethylbromazolam is presented in Figure S5.5.

**Nuclear Magnetic Resonance Spectroscopy (NMR)**

Spectrometer: Bruker AVANCE NEO 400, Probe: Bruker 5 mm BBO with z‐gradient, Sample preparation: approx. 10 to 15 mg of the sample was dissolved in deuterated solvents directly in the NMR tube, Measurement: Measurements were performed with sample spinning, 1D-1H: 400 MHz, pulse program: zg30, number of scans: 16 or more, 30° pulse, spectral width: 14 ppm, transmitter offset: 5.0 ppm, time-domain: 64 k, spectrum size: 64 k, exponential multiplication with line broadening 0.3 Hz, 1D-13C: 100 MHz, pulse program: zgpg30, number of scans: 1024 or more. Peak assignments were corroborated by COSY, HSQC and HMBC. The 1D NMR spectrum of ethylbromazolam is presented in Figures S5.6-S5.9, the COSY NMR spectrum in Figure S5.10, the HSQC NMR spectrum in Figures S5.11-S5.13, the HMBC NMR spectrum in Figures S5.14-S5.15, and an summary of the NMR results in Figure S5.16.

5. ***In vitro* GABA_A_ receptor activity**

**Assay protocols.** The whole-cell protocol used had a holding potential of -90 mV and -20 mbar. The chip validation required a resistance between 0.5 and 10 MΩ. The cell positioning time was 30 sec with a positioning pressure of -100 mbar. For whole-cell, five suction pulses were used starting at a first pulse amplitude of -200 mbar with a -50 mbar increment between pulses, pulse duration of 1000 ms, and a pulse period of 10,000 ms. The cells were clamped to -90 mV throughout the recordings. The minimum seal resistance was1 MΩ, liquid dispense delay of 3000 ms, data acquisition time of 14,000 ms, cut-off frequency of 3000 Hz, sampling frequency of 10000 Hz, and filter order of 8. The signal was filtered at 3000 Hz (filter order 8) using a Bessel filter.

**SECTION 2**

**Data from Toronto’s Drug Checking Service (Canada)**

| **Compound** | **Month checked** | | | | | | | | | | | **Total** |
| --- | --- | --- | --- | --- | --- | --- | --- | --- | --- | --- | --- | --- |
|  | **Nov 2024** | **Dec 2024** | **Jan 2025** | **Feb 2025** | **Mar 2025** | **April 2025** | **May 2025** | **June 2025** | **July 2025** | **Aug 2025** | **Sep 2025** |  |
| Bromazolam | 53 | 45 | 34 | 27 | 26 | 11 | 4 | 6 | 5 | 10 | 12 | 233 |
| Ethylbromazolam | 2 |  | 2 | 12 | 32 | 9 | 5 |  | 2 | 5 | 3 | 72 |
| Desalkylgidazepam | 20 | 22 | 10 | 21 | 13 | 7 |  | 1 | 2 | 5 | 16 | 117 |
| Other benzodiazepine | 12 | 26 | 16 | 25 | 23 | 16 | 10 | 7 | 7 | 10 | 17 | 169 |
| Total samples with a benzodiazepine | 67 | 71 | 52 | 64 | 78 | 36 | 19 | 13 | 14 | 25 | 32 | 471 |
| Total samples tested | 379 | 296 | 347 | 357 | 338 | 225 | 205 | 188 | 264 | 275 | 274 | 3148 |

**Table S2.1.** Benzodiazepines detected in samples submitted to Toronto’s Drug Checking Service between November 2024 and September 2025. As there were some samples where mixtures of benzodiazepines were found, the total samples with a benzodiazepine do not always equate to the sum of the different benzodiazepines detected each month.

**SECTION 3**

**Data from WEDINOS (UK)**

**Table S3.1.** Benzodiazepines detected in samples submitted to WEDINOS between December 2024 and September 2025.

| **Benzodiazepine** | **Dec 2024** | **Jan 2025** | **Feb 2025** | **March 2025** | **April 2025** | **May 2025** | **June 2025** | **July 2025** | **Aug 2025** | **Sep 2025** | **Total** |
| --- | --- | --- | --- | --- | --- | --- | --- | --- | --- | --- | --- |
| Ethylbromazolam |  | 6 | 13 | 12 | 20 | 30 | 18 | 46 | 39 | 48 | 232 |
| Bromazolam | 48 | 36 | 47 | 32 | 34 | 43 | 19 | 25 | 27 | 16 | 327 |
| Alprazolam | 30 | 24 | 30 | 33 | 21 | 32 | 11 | 33 | 24 | 21 | 259 |
| Diazepam | 67 | 70 | 98 | 84 | 74 | 114 | 58 | 120 | 92 | 85 | 862 |
| Etizolam | 6 | 6 | 4 | 8 | 6 | 12 | 3 | 6 | 2 | 6 | 59 |
| Clonazepam | 15 | 7 | 18 | 16 | 5 | 24 | 20 | 39 | 21 | 7 | 172 |
| Flubromazolam |  | 2 |  | 2 |  |  |  |  |  |  | 4 |
| Clobromazolam |  | 1 |  | 1 | 2 | 6 | 7 | 4 | 4 | 5 | 30 |
| Rilmazafone |  |  |  |  |  | 1 |  |  | 1 |  | 2 |
| Chlordiazepoxide |  |  |  |  |  |  |  | 1 |  |  | 1 |
| Clobazam |  |  |  |  |  |  |  | 2 |  |  | 2 |
| Flualprazolam | 1 | 1 |  |  |  | 1 |  |  |  | 2 | 5 |
| Clonazolam | 1 | 2 | 1 |  | 1 |  | 2 |  | 2 | 7 | 16 |
| Desalkylflurazepam |  |  |  |  |  |  | 1 |  |  |  | 1 |
| Desalkylgidazepam |  | 1 |  |  |  |  |  | 1 |  |  | 2 |
| Deschloroetizolam |  |  |  | 1 |  |  |  |  |  |  | 1 |
| Lorazepam | 3 | 2 | 9 | 4 | 10 | 9 | 3 | 8 | 10 | 7 | 65 |
| Midazolam |  |  |  |  |  |  |  | 1 |  | 2 | 3 |
| Nitrazepam | 3 |  | 2 | 2 |  | 5 | 2 | 2 | 4 |  | 20 |
| Phenazepam |  |  |  |  |  | 3 |  | 1 |  |  | 4 |
| Pyrazolam |  |  |  |  |  |  | 1 | 1 |  |  | 2 |
| Rilmazafone |  |  |  |  | 1 | 1 |  |  |  | 1 | 3 |
| Temazepam | 1 |  |  |  |  |  |  |  |  | 1 | 2 |
| Bromazepam |  | 1 |  |  |  |  |  |  |  | 1 | 2 |
| Flubromazepam |  | 1 |  |  |  |  |  |  |  |  | 1 |
| Methylclonazepam |  |  |  |  |  |  |  |  |  | 1 | 1 |
| Total detections | 175 | 160 | 222 | 195 | 174 | 281 | 145 | 290 | 226 | 210 | 2078 |
| Total samples | 174 | 150 | 219 | 188 | 171 | 280 | 142 | 287 | 219 | 212 | 2042 |

**SECTION 4**

**Data from CanTEST (Australia)**

**Table S4.1.** Samples analyzed by CanTEST in Australia found to contain ethylbromazolam, including their date of receipt, form, the drug it was expected to be by the one submitting the sample, the result of the benzodiazepine immunoassay test strip (BTS), and the drugs detected using UPLC-PDA and UPLC-MS or UPLC-MS/MS for the first sample listed.

| **Date** | **Form** | **Expected** | **BTS** | **Detections (UPLC-PDA and UPLC-MS/MS)** |
| --- | --- | --- | --- | --- |
| 2025-04-11 | White Mylan branded pill | Alprazolam (Mylan) | Negative | Ethylbromazolam  Desalkylgidazepam  Other minor components |
| 2025-05-01 | White round Mylan branded pill fragment | Unknown benzodiazepine | Not tested | Ethylbromazolam |
| 2025-05-09 | White Xanax branded bar shaped pill | Alprazolam (Xanax) | Positive | Ethylbromazolam  Alprazolam (minor) |
| 2025-06-06 | Pale yellow round pill with G on one side | Diazepam | Not tested | Ethylbromazolam |
| 2025-08-01 | Round white pill with CN and 2 on one side | Clonazepam | Positive | Ethylbromazolam  Bromazolam  Desalkylgidazepam |
| 2025-08-01 | White Xanax branded bar shaped pill | Alprazolam (Xanax) | Negative | Ethylbromazolam  AB-MDMSBA  *N*-cyclohexylpentylone |
| 2025-08-07 | White Xanax branded bar shaped pill fragment | Alprazolam (Xanax) | Positive | Bromazolam  Ethylbromazolam  MDMA  Related BZO hydrolysis |
| 2025-08-07 | Yellow round pill with DM 5 and G on flip side | Not sure | Negative | Ethylbromazolam  Ethylbromazolam hydrolysis product |

**Figure S4.1** A) UPLC-PDA chromatogram of client sample containing ethylbromazolam. B) PDA spectrum of ethylbromazolam. C) Overlay of bromazolam standard (R.T 3.418 min) and ethylbromazolam (R.T 4.217 min) spectra from UPLC-PDA analysis.

**Figure S4.2** High resolution UPLC-MS/MS spectrum of ethylbromazolam.

**SECTION 5**

**Analytical results of ethylbromazolam sample from ADEBAR (Germany)**

Ethylbromazolam

Caffeine

Oleamide

**Figure S5.1.** GC spectrum of ethylbromazolam from GC-EI-MS analysis.

**Figure S5.2.** EI-MS spectrum of ethylbromazolam from GC-EI-MS analysis.

**Figure S5.3.** GC-sIR spectrum of ethylbromazolam.

**Figure S5.4.** Fingerprint/zoom of GC-sIR spectrum of ethylbromazolam.

**Figure S5.5.** LC-ESI-MS spectra of ethylbromazolam.

**Figure S5.6.** 1D NMR spectrum of ethylbromazolam.

**Figure S5.7.** Zoom-in of 1D NMR spectrum of ethylbromazolam.

**Figure S5.8.** Zoom-in of 1D NMR spectrum of ethylbromazolam.

**Figure S5.9.** Zoom-in of 1D NMR spectrum of ethylbromazolam.

**Figure S5.10.** COSY NMR spectrum of ethylbromazolam.

**Figure S5.11.** HSQC NMR spectrum of ethylbromazolam.

**Figure S5.12.** Zoom-in of HSQC NMR spectrum of ethylbromazolam.

**Figure S5.13.** Zoom-in of HSQC NMR spectrum of ethylbromazolam.

**Figure S5.14.** HMBC NMR spectrum of ethylbromazolam.

**Figure S5.15.** Zoom-in of HMBC NMR spectrum of ethylbromazolam.

**Figure S5.16.** Summary of NMR results of ethylbromazolam.

**SECTION 6**

**Statistical analysis results**

**Table S6.1.** Statistical comparison from Brown-Forsythe and Welch ANOVA tests (α = 0.05) for the efficacy (E_max_) and potency (EC_50_) between diazepam, bromazolam, and ethylbromazolam.

| **Comparisons** | | **Efficacy (E_max_)** | | | **Potency (EC_50_)** | | |
| --- | --- | --- | --- | --- | --- | --- | --- |
| **Compound 1** | **Compound 2** | **Mean Diff** | **P value** | **Significant?** | **Mean Diff** | **P value** | **Significant?** |
| Bromazolam | Diazepam | -0.8344 | 0.0142 | Yes | -0.1626 | 0.8817 | No |
| Ethylbromazolam | Diazepam | -1.224 | 0.0010 | Yes | -0.3401 | 0.5058 | No |
|  | Bromazolam | -0.3897 | 0.1130 | No | -0.1775 | 0.9012 | No |

**SECTION 7**

**Screenshots of Reddit posts on ethylbromazolam**
